# Regul-A: A Technological Application for Sensory Regulation of Children with Autism Spectrum Disorder in the Home Context

**DOI:** 10.3390/ijerph181910452

**Published:** 2021-10-05

**Authors:** Helena Reis, Inês Eusébio, Margarida Sousa, Mariana Ferreira, Raquel Pereira, Sara Dias, Catarina I. Reis

**Affiliations:** 1 ciTechCare, Polytechnic of Leiria, Campus 2—Morro do Lena, Alto do Vieiro, 2411-901 Leiria, Portugal; sara.dias@ipleiria.pt (S.D.); catarina.reis@ipleiria.pt (C.I.R.); 2 School of Health Sciences, Polytechnic of Leiria, Campus 2—Morro do Lena, Alto do Vieiro, Apartado 4137, 2411-901 Leiria, Portugal; euzenis@gmail.com (I.E.); margaridas998@gmail.com (M.S.); m.lf26@hotmail.com (M.F.); raquel-pereira98@hotmail.com (R.P.); 3 School of Management and Technology, Polytechnic of Leiria, Campus 2—Morro do Lena, Alto do Vieiro, Apartado 4163, 2411-901 Leiria, Portugal

**Keywords:** autism spectrum disorder, sensory regulation, home routines

## Abstract

(1) Background: Sensory processing disorder is now recognised as a core feature of autism spectrum disorder that influences children’s adaptive behaviours, which, in turn, may interfere with their participation in life situations. This study describes the process of developing a technological platform, in the form of an app, to help families regulate children with ASD, aged 3–6 years old, by applying sensory strategies to improve the child’s participation in daily routines in the home context. (2) Methods: A focus group formed by four specialised occupational therapists who intervene with children with ASD was selected in order to understand and discuss content that should be included in the app. At a later stage, a group of three was involved to ensure quality and veracity in technological platform elaboration. (3) Results: The purpose of the app, named Regul-A, is to help parents regulate children with ASD regarding their participation in home routines. The sensory strategies provided by the focus group in the three major occupations of the child were the first results obtained, followed by the development of the app structure. (4) Conclusions: The next phase of the study will be the use of the platform by families of children with ASD and occupational therapists. It is believed that, in the future, Regul-A will be used as a tool to gather, analyse and manage data on the occupational performance of children with ASD in the home context, particularly for activities of daily living, sleep, rest and play, facilitating the implementation of strategies and the sharing of information between parents and occupational therapists.

## 1. Introduction

Autism spectrum disorder (ASD) is a neurodevelopmental disorder characterised by persistent impairments in social interaction and the presence of restricted, repetitive patterns of behaviours, interests or activities [1]. About 1 in 54 children have been identified with autism spectrum disorder (ASD), according to estimates from the Centre for Disease Control and Prevention (CDC) and Autism and Developmental Disabilities Monitoring (ADDM) [2]. The recognised, widespread presence of atypical sensory reactivity among people with ASD recently led to its inclusion as a diagnostic feature of ASD in the Diagnostic and Statistical Manual of Mental Disorders (5th ed.) [1]; under the criterion of “restricted, repetitive patterns of behavior, interests, or activities”. Studies estimate that 70–90% of children with autism spectrum disorder (ASD) display atypical reactivity to sensory stimuli [3]. This atypical reactivity is often characterised into three types that regularly coexist in individuals with ASD: hypo-reactivity (decreased or no reaction to sensory stimuli when it would be expected), hyperreactivity (increased or aversive reaction to sensory stimuli) and sensory seeking (behaviours that intensify or extend sensory experiences) [4]. Children who hyper-react to stimuli notice things much more than do others. Because these children notice more, one might observe that they are more isolated than other children and are anxious more quickly than others in the family or at school (e.g., responds negatively to a hair dryer or vacuum cleaner). These children may be more interested in being alone or in very quiet places. When environments are too challenging, these children may withdraw and, therefore, not complete activities in daily life. In general, these children will be better able to participate in everyday life activities when there is less sensory input available in the environment. Children who are hypo-reactive have a more intense response in low registration; this means that they miss more cues than others (i.e., they fail to notice things). Because these children notice less, one might observe that they are more easy-going than other children and are undisturbed by things that others in the family or classroom notice (e.g., rocks unconsciously, spins or seeks all kinds of movement). However, not noticing can also mean that children do not respond when called, may drift away during activities and have a harder time completing tasks in a timely manner. In general, these children can profit from adults providing more intense sensory experiences that are naturally integrated into the routines of their daily life. With more intensity of sensory input, these children can pay attention for a longer time during daily life activities.

Hypo-reactivity has most consistently been reported in young children with or at risk for ASD [5], though hyperreactivity and sensory seeking are also regularly reported [3,4,6]. Detailed examination of the nuances of these hypo- and hyperreactivities and seeking patterns has suggested that certain aspects of auditory processing, lower registration or modality sensitivities (e.g., tactile, oral, touch) may influence these atypical reactivity patterns [7]. These atypical sensory reactions suggest dysfunctional sensory processing in the central nervous system and can explain the compromises in terms of attention, arousal, affect and action—called the four As—in terms of regulating the child’s behaviour [8].

Problematic responses to everyday situations in children with ASD are thought to represent an interaction between child cognitive factors and triggers in the environment to which the child is sensitive. Child factors include poor social awareness/abnormalities in social–information processing, heightened arousal or emotional dysregulation [9], rigidity [10], intolerance of uncertainty [11] and sensory sensitivities [3]. Vulnerabilities manifest in triggering environmental contexts, e.g., when feared stimuli are present [12], in the context of routine demands [13], in the absence of parental/teacher attention/provision of specific activities [10], when there are changes in routines/environments or when things are not “on the child’s terms” [14]. Families report that behaviours associated with difficulty processing and integrating sensory information create social isolation for them and their child, restrict participation in daily living activities and impact social engagement [4]. Moreover, teachers do not feel competent or confident when teaching students with ASD [15], and truly including these students within the mainstream classroom seems a challenging task [16]. Due to the heterogeneity in the population, it is difficult for educational professionals to meet the diverse needs of these students with ASD [17].

Some people with autism gravitate towards technology for learning, play and communication. For many, technology in the form of augmented communication aids has helped to give them a voice. Moreover, many families are becoming increasingly disillusioned with autism research. Many feel that research outcomes have become distanced from practical strategies that help families manage the challenges that come with autism [18]. There has been extensive research attempting to demonstrate the efficiency of technologies as support tools for children with ASD [19]. Interestingly, the highest percentage rely on conceptual skills, practical skills, social skills and general skills. Studies that focus on social skills are predominant. Researchers believe that technologies that offer safe, interactive and therapeutic environments will only come about from a multidisciplinary team of clinicians, software developers, people on the autism spectrum and their families [18].

Therefore, there is a gap in the research regarding technologies that help parents and professionals regulate these children when they are in need and in specific contexts.

In solving this challenge, this research aimed to promote the development of an innovative and adaptative technological platform, Regul-A, in which the sensory profile of the child with ASD is identified (hypo- or hyperreactive) in the different sensory systems (auditory, visual, tactile, olfactory, gustatory, proprioceptive and vestibular).

The platform will include several sensory strategies that aim to regulate the child in their daily activities (routines) and improve their participation and functionality in the home context. It is also intended that through this platform, strategies can be adapted or added by the different professionals who work with the child and parents. This will allow better communication between all and, consequently, lead to more efficient participation of the child in their home routines. Regul-A aims to target the child, resulting in a more regulated, participative and functional child with ASD.

## 2. Materials and Methods

This study was approved by the Ethics Committee of the Polytechnic of Leiria (CE/IPLEIRIA/31/2020). The research used a qualitative methodology and is classified as a descriptive and cross-sectional study. In this phase of the study, the intention was to (1) identify the daily participation routines of the child with ASD in the home context, (2) establish sensory strategies used to regulate the sensory reactivity of a child with ASD and (3) develop an mHealth platform to support the use of appropriate sensory strategies for children aged 3–5 years with ASD.

For the first and second objectives, a focus group of occupational therapists was selected for the fact that their expertise and interaction with participants added value to the data. For the inclusion criteria, occupational therapists were required to have at least five years of experience in early intervention with children with ASD and their families and specialised training in sensory integration theory and practice.

The researchers contacted three autism associations that work in a specialised and qualified way in the intervention of children between 0–6 years old with ASD and also the clinical directors of five private paediatric clinics in the north of the country. The focus group was formed by one occupational therapist of an autism association and three occupational therapists from three private paediatric clinics. One of these occupational therapists was the mother of a child with a sensory processing disorder, and so her expertise associated with her experience as a mother of a special child was an asset in this research.

Informed consent for participation in the research was signed by the participants of the focus group, in accordance with the Declaration of Helsinki and The Oviedo Convention, in which permission was requested for audio recording during the course of the various focus group sessions, which was used in subsequent data analysis.

For the third objective, the development of the app, in collaboration with the Computer Science course of the School of Technology and Management of the Polytechnic of Leiria was established so that, through a multidisciplinary team, a more consistent app could be created, bringing together knowledge of health and informatics and allowing better quality and accuracy.

### 2.1. Focus Group

A semi-structured interview script was prepared with the aim of creating a guideline for sharing ideas on the theme, with the aim of understanding the content that the app should include by integrating the participants’ perspectives. This instrument was chosen because it was flexible and adapted to the participants, their context and the course of the discussion. The script consisted of open-ended questions that defined the occupation area that should be investigated.

According to Occupational Therapy Practice Framework (OTPF) 4th edition [20], occupations are central to a client’s (person’s, group’s, or population’s) health, identity and sense of competence and have particular meaning and value to that client; that is, “in occupational therapy, occupations refer to the everyday activities that people do as individuals, in families, and with communities to occupy time and bring meaning and purpose to life. Occupations include things people need to, want to and are expected to do” [21] (para. 2). In the OTPF–4, the term occupation denotes personalised and meaningful engagement in daily life events by a specific client. When occupational therapy practitioners work with clients, they identify the types of occupations clients engage in individually or with others. Differences among clients and the occupations they engage in are complex and multidimensional. Caregiving is a co-occupation that requires active participation by both the caregiver and the recipient of care. For the co-occupations required during parenting, the socially interactive routines of eating, feeding and comforting may involve the parent, a partner, the child and significant others [22].

To obtain sensory strategies for all the children’s occupations in the home context by the focus group, we based the semi-structured interview on the occupations presented in the 4th edition of OTPF. This categorisation was also supported by the Division for Early Childhood Recommended Practices, which also recommend that practitioners who work with the family and other adults should modify and adapt the physical, social and temporal environments to promote each child’s access to and participation in learning experiences.

Although the script had been prepared prior to the focus group, it underwent changes throughout the process, according to the suggestions and different perspectives of the occupational therapists involved. Four discussion events with the focus group were held between December 2020 and January 2021, with an estimated duration of one hour and thirty minutes each. These discussions took place online due to the restrictions imposed by the COVID-19 pandemic, though it should be noted that there was always an environment conducive to the discussion of different perspectives and the sharing of experiences, contributing to a greater richness of data.

Each discussion event was dedicated to a different topic. The first focused on the constitution and relevance of the app, the sensory systems and also the issues of sensory modulation and reactivity, which allowed for the identification of relevant profiles for the app. The second focused on the selection of occupational areas and relevant daily home activities according to the child’s needs, the definition of the app users, as well as the contexts to which the child belongs, which should be included in the app. The third and fourth meetings consisted of outlining sensory strategies to improve the child’s occupational performance. Furthermore, the organisation of the app and the provision of feedback from parents regarding the strategies were discussed.

The environmental strategies the focus group addressed encompassed the physical environment (e.g., space, equipment and materials), the social environment (e.g., interactions with peers, siblings and family members) and the temporal environment (e.g., sequence and length of routines and activities) on the occupations selected (see Table 1) according to hyper- or hypo-reactivity profile.

Throughout the data collection process, the existing literature on children with ASD and strategies to regulate children with SPD was searched to substantiate and complement the provided information.

The analysis and processing of the data collected through the focus groups occurred between January and February 2021. The data collected through the qualitative methodology were fully transcribed from the audio recordings and subsequently interpreted and organised to obtain the intended data. The analysed information served as a basis for establishing the sensory and regulatory strategies contained in the app.

### 2.2. Technological Application Process

To discover pre-existing apps related to the theme of the study and explore aspects that could be further innovated, mobile apps were searched for in the Apple and Google Play stores. For this search, the following terms were used: “autism”, “sensory processing”, “sensory regulation”, “sensory integration”, “autism strategies”, “routines” and “parental support”. Thus, the following applications were found: “MyAutismTeam”, which consists of an app composed of a support group for parents of children with ASD, in which they can obtain emotional support and practical tips on treatments or therapies; “Breathe, Think, Do with Sesame”, which is aimed at children aged 2–5 years old and has the objective of developing personal skills such as problem-solving, persistence in activities and overcoming stressful situations; “Autism Help”, which is aimed at parents of children with ASD as a tool to help the child perform daily tasks through specific activities; and “Sensory Treat”, which helps caregivers to adhere to the intervention programme in the home context and also to manage the sensory routines of children with SPD, namely in sensory diets. The latter can be set up by occupational therapists for adapting sensory routines to the child’s individual needs. In this sense, the app under development aims to offer a more specific response to parents of children with ASD in order to improve the child’s regulation and participation in their activities in the home context through strategies appropriate to the child’s sensory reactivity.

For the development of the technological platform a protocol was established with the added benefit of participation by a professor of the Computer Science department, who accompanied and guided the investigation together with her group of engineering students. Throughout the process, eight fortnightly meetings lasting an estimated one and a half hours each were held to discuss the construction of the app, with the guiding professors and students from both courses present. These meetings took place online due to the restrictions imposed by the COVID-19 pandemic.

The first meeting aimed to present the PhD professors of both courses (occupational therapy course and informatics engineering course) and the respective students involved, and a brainstorming session was held regarding the main aspects of the app’s structure. Furthermore, it was established that the app would be available for the Android system since it is easier for programming, has a larger number of users and its devices are more affordable. The second meeting focused on the presentation and discussion of the initial prototype of the main menu and also on rating scales regarding feedback on the child’s occupational performance during the family routine. In the third and fourth meetings, the layout and use of graphic elements were discussed in order to provide a more appealing and adequate user experience related to the theme in question, and a customisation option developed by the research group was chosen. In addition, the features of the app’s functions were discussed, namely the Forum, the Weekly Record, the Strategies Feedback, the Favourite Strategies Folder and the Support Chat. In the fifth meeting, the app was tested by the group members, and its name and logo were defined. In the sixth meeting, the tests continued, and the dashboard functionalities were discussed. In the seventh meeting, tests were performed again, the “backoffice” structure was discussed, the layout colour palette was finalised (according to the predominant colours of the logo) and the functionalities to be introduced in the Forum were discussed. Finally, in the last meeting, the “backoffice” functionalities were discussed, as well as adjustments to the app, to conclude the first phase of the research.

Android Studio 4.1.3 (JetBrains, Prague, Czech Republic) and Google Android API 27 version 8.1 (Google, California, USA) were used to build the app. The programming languages used were Android Java for the mobile app and Laravel—PhP for the web application. The app went through alpha testing, going through a testing process by the research group, and will be released as a final version at a later stage after beta testing with real users.

The use of the app requires internet access to log in and access the app, offering a vertical mode user experience, avoiding rotation readjustment and presenting no costs to the user. The login is based on a simple and multi-tenant system so that a user with more than one child may have access to the corresponding profiles without logging out. Users are also allowed to access their accounts on several devices simultaneously. For the development of the app, a user-centred approach was used, with a written and visual language suitable for the user and a practical and intuitive design in order to facilitate usability and the user experience. Regarding the security of the data input into the app, it is guaranteed that no sensitive information, namely the login and password data, are compromised, as it is not stored in plain text and is hash encrypted.

## 3. Results

### 3.1. Application Content

Through the focus group meetings, it was possible to define the daily participation routines of the child with ASD and establish the sensory strategies for regulating the sensory reactivity of a child with ASD that should be included in the app.

The sensory profiles to be included were defined according to sensory reactivity, namely hyporeactive or hyperreactive, in order to guide parents in the application of the most appropriate strategies to the child’s sensory needs. The app included a page regarding the child’s profile and sensory needs according to each sensory system (olfactory, gustatory, visual, auditory, tactile, vestibular and proprioceptive), and this information should be entered by the occupational therapist. The sensory strategies were assigned to the occupations, namely activities of daily living (dressing, bathing and showering, eating and feeding, personal hygiene and grooming, toileting and toilet hygiene), sleep and rest and play since they represent frequent occupations in the family routines and those in which parents show more difficulties in regulating the child [19]. A parameter focused on regulatory strategies common to the profiles and the various activities was included, both for the preparation and for the activity itself, allowing for better regulation and, consequently, participation of the child. A feedback space was also included so that parents could validate the implementation and results of the strategies in the child’s occupational performance.

### 3.2. Application Structure

Regul-A was chosen as the name for the app since its purpose is to regulate (“Regul”) children with autism (“A”) during their home activities.

It was possible to build Regul-A based on the following aspects:

The occupational therapist uses the web app (backoffice) to manage the information available to parents, namely the evaluation and identification of the child’s sensory reactivity according to each system, to adapt and specify the strategies according to the child’s sensory needs and to visualise the child’s evolution through the feedback provided by parents.

Through the mobile app, parents can access information and sensory strategies and can also consult their child(ren)’s profile without logging out of their account by accessing the functionality “Change Child Profile”.

The main menu includes the functionalities “Profile”, “Strategies”, “Support Chat”, “Forum”, “Weekly Register” and “Favourite Strategies”.

The Profile contains information concerning the child, namely their name, date of birth, a real or representative image, the child’s sensory reactivity according to each sensory system and comments added by the occupational therapist accompanying the child. In addition, it is possible to access the dashboard functionality, where the child’s performance evolution chart is shown according to the records made on a weekly basis. When creating the profile, parents need to identify the child’s name, date of birth and gender, in addition to the username.

The “Strategies” are previously defined and subdivided, and it is possible to access the lists according to the occupations, select favourite strategies, provide feedback according to the strategies used and access the “Strategy Area”.

The “Feedback” allows the user to note if they were able to use the strategy, evaluating it on a qualitative ordinal scale of three categories, which are colour coded (bad, indifferent and good) and also to add comments. It should be noted that, through the “backoffice”, the occupational therapist has access to all the feedback. The “Strategy Area” contains information regarding the feedback provided, specifically, the number, the average, the last feedback entry and the history of comments.

The “Support Chat” allows the parents and occupational therapists to have direct contact, facilitating effective communication between them. It also provides direct and specialised support in the area, as there is an opportunity to clarify doubts and request relevant indications in the process.

The “Forum” represents a sharing space between the whole app community. With this functionality, it is intended to create dynamics and interaction based on publications about themes related to family routines and sensory strategies to be adopted. In this option, it is possible to create and reply to publications, share attachments and view the most commented publications.

The “Weekly Record” allows parents to evaluate the child’s participation in the occupations according to a 5-point Likert Scale, in which 1 corresponds to “Did not participate at all” and 5 corresponds to “Participated fully”. For example, parents could answer a question, such as “During the last week, how would you evaluate your child’s participation in the following activities: bathing and showering, dressing, eating and feeding, personal hygiene and grooming (brushing teeth, cutting nails, washing hands, applying cream, cutting hair), toileting and toilet hygiene, sleep and rest, play”.

Through the “Favourite Strategies” folder, it is possible to access the strategies that best suit the child’s sensory needs, regardless of the area of occupation, in order to facilitate subsequent consultations. They can be added or removed from the folder at any time.

Regarding the presentation and layout of the app, these were adapted to the target population in order to optimise the user experience. In this sense, images alluding to the theme of the app were created, using a varied and contrasting colour pattern so as to facilitate the representation of the child, recognition of the various sensory systems, identification of the respective activities and occupations and also the search for strategies.

All graphic elements that make up the app were analysed in order to obtain greater visual consistency and ease in locating the available information and, consequently, enhance the app’s usability. Similarly, linguistic elements were considered in order to promote correct interpretation by the parents through a direct, simple and objective language, also resorting to the exemplification of concrete situations.

## 4. Discussion

This study provides a better understanding of the content and structure of the Regul-A app. This app was developed to facilitate the regulation of a child with ASD at home, as well as to be a tool to gather, analyse and manage data on the child’s occupational performance, facilitating the implementation of strategies and the sharing of information between parents and occupational therapists.

During the construction process, it was possible to identify the following strengths: The collaboration of occupational therapists with experience in the area of conducting a focus group, which allowed for the identification and adequation of sensory strategies according to the daily occupations of the child and sensory reactivity profiles, respectively. The results presented by the focus group became essential in developing the app’s theoretical and practical approach. The partnership with the Department of Computer Science proved to be crucial for the design of the app, with sharing of knowledge by the professions involved, which provided a cooperative and enriching environment. The Regul-A app is an innovative and distinct tool since there is currently no product with similar characteristics on the market, allowing it to offer relevant and unique content.

During the construction of Regul-A, the app underwent alpha testing by an occupational therapist with vast experience in working with children with ASD, an informatics engineer and a biostatistician (someone outside the field of autism spectrum and informatics for improved and impartial analyses) in order to identify errors in the app prototype and to make improvements in its functionalities, language and layout.

Regarding the use of the app by occupational therapists, the “backoffice” is considered a facilitating and complementary tool for intervention with the following relevant aspects: remote monitoring of the child, an indication of strategies according to sensory needs since these are already presented for the home context, continuous interaction with the child’s parents, greater sharing of information with parents, closer contact with the real difficulties experienced in the home context and evolution of the child’s participation in home routines.

Due to the fact that parents are the primary contact with the child, it became relevant for Regul-A to create opportunities for parents to interact as a group and share experiences through the “Forum” functionality.

It is important to highlight the importance of developing strategies that help parents in their daily routines since they may not have the knowledge to meet the sensory demands of their children. In this sense, Regul-A can be a solution providing reliable and personalised information to parents through specific sensory strategies that they can apply in their context, promoting learning and development of new skills of the child.

A future direction for research is a pilot study, with the main objective of assessing the applicability of the Regul-A for testing and making the necessary modifications according to recommendations by 10 parents of children with ASD (3–5 age) and their occupational therapists.

## 5. Conclusions

The great purpose of this study was achieved: to build an app that can help parents of children with autism regulate their children in daily living activities in the home context.

Through the focus group, the daily routines of a child with ASD in the home context were identified, and the sensory reactivity of children with ASD (hypo- and hyperreactivity in different sensory systems) was classified across several defined home routines. They determined sensory strategies that, when used, could help regulate the reactivity of a child with ASD (hypo- or hyperreactivity) in the home context, thus decreasing dysregulatory behaviours and improving their participation in different activities and routines.

Together with the informatic engineering team, the technological mHealth platform was designed according to the information/data collected with the focus group to support the better participation of these children in the home context. We also developed and integrated two major components of the mHealth platform (app and backoffice) and conducted preliminary tests over the entire system. We believe that future studies should consider and validate the use of Regul-A in the home context.

Further research, including school and community contexts, is necessary since the child is integrated with other natural contexts with other specific sensory characteristics. Due to the current pandemic situation, changes had to be made at the research level, and it was not possible to apply the second phase of this research—a pilot study—with the main objective of assessing the applicability of the app by parents and occupational therapists and, consequently, testing and making the necessary modifications; however, this will be the next step.

The results presented by the focus group became essential to develop the theoretical and practical approach of the app, and the partnership with the Department of Computer Science proved to be crucial for the design of the app, with sharing of knowledge by the professions involved, which provided a cooperative and enriching environment. Research has focused on supporting children with ASD by using technologies such as virtual reality, augmented reality, virtual agents, sensors and geolocation through educational games but has not focused on families’ needs.

The Regul-A app seems to be an innovative and distinct tool since there is currently no product with similar characteristics on the market, allowing it to offer relevant and unique content. We think that the use of technologies in conjunction with families’ needs, users’ experiences and accessibility design and evaluation are promising research topics related to regulating children with ASD. With this study, we intend to develop this app.

## Figures and Tables

**Table 1:** Children’s occupations in home context are categorised as activities of daily living, rest and sleep and play (adapted from OTPF 4th ed).

Occupation	Description
Activities of daily living (ADLs)—activities oriented towards taking care of one’s own body and completed on a routine basis (adapted from Rogers and Holm [23])	
Bathing, showering	Obtaining and using supplies; soaping, rinsing and drying body parts; maintaining bathing position; transferring to and from bathing positions
Dressing	Selecting clothing and accessories with consideration of time of day, weather and desired presentation; obtaining clothing from storage area; dressing and undressing in a sequential fashion; fastening and adjusting clothing and shoes; applying and removing personal devices, prosthetic devices or splints
Toileting and toilet hygiene	Obtaining and using toileting supplies, managing clothing, maintaining toileting position, transferring to and from toileting position, cleaning body, caring for menstrual and continence needs (including catheter, colostomy and suppository management), maintaining intentional control of bowel movements and urination and, if necessary, using equipment or agents for bladder control
Eating and swallowing	Keeping and manipulating food or fluid in the mouth, swallowing it (i.e., moving it from the mouth to the stomach)
Feeding	Setting up, arranging and bringing food or fluid from the vessel to the mouth (includes self-feeding and feeding others)
Personal hygiene and grooming	Obtaining and using supplies; removing body hair (e.g., using a razor or tweezers); applying and removing cosmetics; washing, drying, combing, styling, brushing and trimming hair; caring for nails (hands and feet); caring for skin, ears, eyes and nose; applying deodorant; cleaning mouth; brushing and flossing teeth; removing, cleaning and reinserting dental orthotics and prosthetics
Rest and sleep—activities related to obtaining restorative rest and sleep to support healthy, active engagement in other occupations	
Rest	Identifying the need to relax and engaging in quiet and effortless actions that interrupt physical and mental activity; reducing involvement in taxing physical, mental or social activities, resulting in a relaxed state; engaging in relaxation or other endeavours that restore energy and calm and renew interest in engagement
Sleep preparation	Engaging in routines that prepare the self for a comfortable rest, such as grooming and undressing, reading or listening to music, saying goodnight to others and engaging in meditation or prayers; determining the time of day and length of time desired for sleeping and the time needed to wake; establishing sleep patterns that support growth and health (patterns are often personally and culturally determined); preparing the physical environment for periods of sleep, such as making the bed or space on which to sleep, ensuring warmth or coolness and protection, setting an alarm clock, securing the home (e.g., by locking doors or closing windows or curtains), setting up sleep-supporting equipment (e.g., CPAP machine) and turning off electronics and lights
Sleep participation	Taking care of personal needs for sleep, such as ceasing activities to ensure onset of sleep, napping and dreaming; sustaining a sleep state without disruption; meeting night-time toileting and hydration needs, including negotiating the needs of and interacting with others (e.g., children, partner) within the social environment, such as providing night-time caregiving (e.g., breastfeeding) and monitoring comfort and safety of others who are sleeping
Play—activities that are intrinsically motivated, internally controlled, and freely chosen and that may include suspension of reality (e.g., fantasy), exploration, humour, risk-taking, contests and celebrations [24]; play is a complex and multidimensional phenomenon that is shaped by sociocultural factors [25]	
Play exploration	Identifying play activities, including exploration play, practice play, pretend play, games with rules, constructive play and symbolic play
Play participation	Participating in play; maintaining a balance of play with other occupations; obtaining, using and maintaining toys, equipment and supplies

## Data Availability

No data other than that shown in the manuscript have been reported.
